# Supplementary material for: Efficacy of Treatment for Metastatic Hormone-Sensitive Prostate Cancer: An Umbrella Review of Systematic Reviews and Meta-Analyses
Source: Cancers (Basel). 2023 Dec 6;15(24):5714. doi: 10.3390/cancers15245714 (PMC10742269; doi:10.3390/cancers15245714)
Supplement: Supplementary file 1 [file cancers-15-05714-s001.zip › cancers-2715426-supplementary.pdf]

Supplementary Materials

Supplement Table 1. Search term and strategies

Supplement Table 2. Excess significant test reported OS outcome in meta-analysis

Supplement Table 3. Citation matrix of all included systematic reviews and meta-analysis and Overlapping test for all reported outcomes

Supplement Table 4. Citation matrix of all included systematic reviews and meta-analysis and Overlapping test for OS outcome

Supplement Table 1. Search term and strategies

PUBMED

P	Search term	Number
#1	“Prostatic Neoplasms” [MeSH Terms]	128,139
#2	(advance*) OR ("Neoplasm Metastasis"[MeSH Terms])	1,276,026
#3	((("hormone dependent") OR "hormone naive") OR "hormone sensitive") OR (("castration sensitive") OR ("castration naive"))	16,072
#4	#1 AND #2 AND #3	975

I	Search term	Number
#5	orchiectomy	18,279
#6	((("Gonadotropin-Releasing Hormone antagonist*") OR ("GnRH antagonist*")) OR ("LHRH antagonist*")) OR (((("GnRH agonist*") OR ("LHRH agonist*")) OR ("Gonadotropin-Releasing Hormone agonist*)) OR ((LHRH) OR (GnRH)))	45,073
#7	Buserelin	2462
#8	Goserelin	1899
#9	Leuprolide	3427
#10	Triptorelin	2156
#11	(firmagon) OR (Degarelix)	244
#12	abarelix	62
#13	relugolix	17
#14	Docetaxel	16,670

#15	Enzalutamide	1,944
#16	Abiraterone	2,263
#17	(Abiraterone) OR (Abiraterone Acetate[MeSH Terms])	2,263
#18	Apalutamide	181
#19	#5 OR #6 OR #7 OR #8 OR #9 OR #10 OR #11 OR #12 OR #13 OR#14 OR #15 OR #16 OR #17 OR #18	80,846

O	Search term	Number
#20	survival[MeSH Terms]	4,725
#21	Mortality[MeSH Terms]	382,516
#22	"Progression-Free Survival"	45,865
#23	testosterone	102,547
#24	Quality of Life	333,280
#25	("patients reported outcome") OR ("patient-reported outcomes")	13306
#26	(toxicity) OR (toxicit*)	897,547
#27	"adverse event*"	160,807
#28	Drug-Related Side Effects and Adverse Reactions[MeSH Terms]	115,950
#29	#20 OR #21 OR #22 OR #23 OR #24 OR #25 OR #26 OR #27 OR #28 OR#29	1,987,912

#30	meta-analysis	190,361

Combine P I O S	
#4 AND #19 AND #29 AND #30	1,304

SCOPUS

P	Search term	Number
#1	("Prostate cancer") OR ("Prostatic cancer")	642,253
#2	(metastasis OR metastatic) OR (advanced OR advance) OR (stage4)	14,572,580

#3	"castration resistant"	37,759
#4	#1 AND #2 NOT #3	408,404

I	Search term	
#5	(orchiectomy OR orchidectomy)	24,563
#6	(buserelin OR goserelin OR leuprolide OR triptorelin OR "Lut einizing hormone-releasing hormone" OR lhrh OR "Gonadotropin-Releasing Hormone" OR gnrh)	55,357
#7	(firmagon OR degarelix OR abarelix OR relugolix OR "Lutein izing hormone-releasing hormone antagonist" OR "lhrh antagonist" OR "Gonadotropin-Releasing Hormone antagonist" OR "gnrh antagonist")	4,612
#8	(docetaxel)	49,112
#9	(enzalutamide)	3,768
#10	(abiraterone)	4,550
#11	(apalutamide)	382
#12	#5 OR #6 OR #7 OR #8 OR #9 OR #10 OR #11	128,769

O	Search term	Number
#13	(survival)	1,693,733
#14	("Progression-Free Survival")	74,635
#15	(testosterone)	146,211
#16	("quality of life")	510,400
#17	(toxicities OR "adverse events")	969,522
#18	#13 OR #14 OR #15 OR #16 OR #17	3,083,500

S	Search term	Number
#19	"systematic review" OR meta-analysis	4,039,383

Combine P I O S	
#4 AND #12 AND #18 AND #19	2,831

Supplement Table 2. Excess significant test reported OS outcome in meta-analysis

MA id/ Author	(year)	Comparison	Outcome	Total Number of Primary studies (publication)	Number of primary trials	Pooled effects (95% CI)	Excess significance	
							O/E	*p-value*
1	2021	Mixed ADT vs Docetaxel	OS	7	3	0.80(0.71, 0.89)	2/1.4	0.488
2	2021	Mixed ADT vs Docetaxel	OS	18	3	0.79(0.71,0.89)	2/1.792	0.807
		Mixed ADT vs Abiraterone			2	0.61(0.54, 0.70)	2/1.997	0.950
		Mixed ADT vs Apalutamide			1	0.67(0.51, 0.89)	1/0.094	0.794
		Mixed ADT vs Enzalutamide			2	0.81(0.53,1.24)	1/1.028	0.968
4	2020	Mixed ADT vs Docetaxel	OS	10	3	0.81 (0.72, 0.92)	2/1.792	0.806
		Mixed ADT vs Abiraterone			2	0.69 (0.61, 0.79)	2/1.997	0.950
		Mixed ADT vs Apalutamide			1	0.64 (0.47, 0.86)	1/0.936	0.794
		Mixed ADT vs Enzalutamide			1	0.53 (0.37, 0.75)	1/1.028	0.968
5	2020	Mixed ADT vs Docetaxel	OS	11	3	0.74 (0.61, 0.90)	2/1.792	0.806
		Mixed ADT vs Abiraterone			3	0.62 (0.53, 0.71)	3/2.994	0.938
		Mixed ADT vs Apalutamide			1	0.63 (0.47, 0.84)	1/0.936	0.794
		Mixed ADT vs Enzalutamide			2	0.65 (0.43 ,0.98)	1/1.028	0.968

		Docetaxel vs Abiraterone			1	1.13 (0.77, 1.66)	0/0.091	0.752
7	2020	Mixed ADT vs Docetaxel	OS	11	3	0.74 (0.66, 0.82)	2/1.792	0.806
		Mixed ADT vs Abiraterone			2	0.62 (0.53, 0.71)	2/1.994	0.938
8	2020	Mixed ADT vs Abiraterone	OS	10	2	0.64 (0.56, 0.73)	2/1.999	0.975
9	2019	Mixed ADT vs Docetaxel	OS	9	2	0.86 (0.69, 1.07)	2/1.710	0.560
		Mixed ADT vs Abiraterone			2	0.85 (0.67, 1.08)	2/1.994	0.938
10	2018	Mixed ADT vs Docetaxel	OS	6	3	0.77 (0.68, 0.88)	2/1.792	0.806
		Mixed ADT vs Abiraterone			2	0.62 (0.53, 0.71)	2/1.994	0.938
12	2018	Mixed ADT vs Docetaxel	OS	5	3	0.75 (0.63, 0.91)	2/1.792	0.806
		Mixed ADT vs Abiraterone			2	0.63 (0.55, 0.72)	2/1.994	0.938
13	2018	Mixed ADT vs Docetaxel	OS	7	3	0.75 (0.65, 0.86)	2/1.792	0.806
		Mixed ADT vs Abiraterone			2	0.63 (0.55, 0.72)	2/1.994	0.938
14	2018	Mixed ADT vs Docetaxel	OS	11	3	0.74 (0.63, 0.86)	2/1.792	0.806
		Mixed ADT vs Abiraterone			2	0.60 (0.50, 0.71)	2/1.994	0.938
16	2017	Mixed ADT vs Abiraterone	OS	2	2	0.62 (0.53, 0.71)	2/1.994	0.938
17	2016	Mixed ADT vs Docetaxel	OS	6	3	0.75 (0.61, 0.91)	2/1.739	0.760
18	2016	Mixed ADT vs Docetaxel	OS	5	2	0.73 (0.64, 0.84)	1/1.086	0.903
21	2016	Mixed ADT vs Docetaxel	OS	2	2	0.73 (0.59, 0.90)	2/1.739	0.584
22	2016	Mixed ADT vs Docetaxel	OS	3	3	0.77 (0.68, 0.87)	2/2.016	0.984

Supplement Table 3. Citation matrix of all included systematic reviews and meta-analysis and Overlapping test for all reported outcomes

Individual study		SRMAs																										
		2021	2021	2021	2020	2020	2020	2020	2020	2019	2018	2018	2018	2018	2018	2017	2017	2016	2016	2016	2016	2016	2016	2016	2015	2019	2017	2016
		Ferro, M.	Wang, L.	Abufaraj, M.	Sathianathen, N.J.	Chen, J.	Wang, Y.	Nunno, V.D.	Sathianathen, N.J.	Landre, T.	Vale, C.L.	Feyerabend, S.	Wallis, C.J.D.	Kassem, L.	Tan, P.S.	Aoun, F.	Rydzewska, L.H.M.	Ramos-Esquivela, A.	Botrel, T.E.A.	Hosseini, S.A.	Lei, J.H.	Tucci, M.	Vale, C.L.	Sciarra, A.	Frank Kunath	Zhu, X.	Zhu, J.	Scailteux
Hoyle, A. P.	2019	⊙	⊙			⊙			⊙																			
Sydes, M. R.	2018	⊙	⊙			⊙						⊙																
James, N. D.	2017		⊙		⊙	⊙	⊙	⊙	⊙	⊙	⊙	⊙	⊙	⊙	⊙	⊙	⊙										⊙	
Kyriakopoulos, C. E.	2018	⊙	⊙					⊙				⊙																
Gravis, G.	2016	⊙	⊙		⊙	⊙	⊙	⊙			⊙	⊙	⊙	⊙	⊙	⊙			⊙									
Clarke, N. W.	2019	⊙	⊙				⊙																					
James, N.	2020	⊙																										
Fizazi, K.	2019	⊙	⊙		⊙		⊙		⊙																			
Chi, K. N.	2019	⊙	⊙		⊙	⊙	⊙	⊙																				
Davis, I. D.	2019	⊙			⊙	⊙	⊙	⊙																		⊙		

Gravis, G.	2013		⊙		⊙	⊙		⊙						⊙	⊙			⊙	⊙		⊙	⊙	⊙					
Sweeney, C. J.	2015		⊙		⊙	⊙	⊙	⊙		⊙		⊙	⊙	⊙	⊙	⊙		⊙	⊙			⊙	⊙					
Morgans, A. K.	2018		⊙					⊙																				
James, N. D.	2016		⊙		⊙	⊙		⊙		⊙	⊙	⊙	⊙	⊙	⊙	⊙		⊙					⊙					
Fizazi, K.	2017		⊙		⊙	⊙		⊙	⊙	⊙	⊙	⊙	⊙	⊙	⊙	⊙	⊙										⊙	
Chi, K. N.	2018		⊙																									
Fukasawa, S.	2018		⊙																									
Agarwal, N.	2019		⊙																									
Armstrong, A. J.	2019		⊙		⊙	⊙	⊙	⊙																		⊙		
Davis, I. D.	2019		⊙																									
Klotz, L.	2008			⊙																⊙				⊙				⊙
Anderson, J.	2013			⊙																				⊙				
Mason, M.	2013			⊙																⊙				⊙	⊙			
Axcrona, K.	2012			⊙																⊙				⊙				
Tombal, B.	2012			⊙																								
Shore, N.	2016			⊙																								
Celestia S. H.	2015			⊙																								
Ozono, S.	2018			⊙																								
Margel, D.	2019			⊙																								

Fizazi, K.	2018								⊙																			
Fizazi, K.	2013								⊙																			
Fizazi, K.	2017								⊙																			
James, N.D.	2017								⊙																			
Fizazi, K.	2018								⊙																			
Sweeney, C.	2016										⊙																	
Gravis, G.	2018											⊙																
Patrick-Miller, L.J.	2016											⊙																
Sweeney, C.	2014																		⊙		⊙							
Sydes, M. R.	2017											⊙																
Rajan, P.	2015													⊙														
Gravis, G.	2015																		⊙			⊙	⊙					
James, N.D.	2015																		⊙			⊙						
James, N.D.	2015																		⊙									
Tombal, B.	2010																							⊙	⊙			
Lee, D.	2015																							⊙				
Smith, M. R.	2010																							⊙				
Scher, H. I.	2012																									⊙	⊙	
Beer, T. M.	2014																									⊙	⊙	

Penson, D. F.	2016																									⊙	⊙	
Shore, N. D.	2016																									⊙	⊙	
Hussain, M.	2018																									⊙		
Waymont, B.	1992																											⊙
Lukkarinen, O.	1994																											⊙
Robinson, M. R.	1995																											⊙
Chang, A.	1996																											⊙
Mikkola, A. K.	1998																											⊙
Schröder, F. H.	2004																											⊙
Smith, M. R.	2010																											⊙
Aro, J.	1993																											⊙
Iversen, P.	1993																											⊙
Vogelzang, N. J.	1995																											⊙
Zalcberg, J. R.	1996																											⊙
Bono, A. V.	1998																											⊙
de Voogt, H. J.	1998																											⊙
Efstathiou, J. A.	2008																											⊙
Roach, M.	2008																											⊙

Calais da Silva, F. E.	2009																											⦿
Crook, J.	2009																											⦿
Bolla, M.	2010																											⦿
Denham, J. W.	2011																											⦿
Horwitz, E. M.	2008																											⦿
Akaza, H.	2009																											⦿
Akaza, H.	2003																											⦿
Anderson, J.	2013																			⦿					⦿			⦿
Ansari, M. S.	2004																											⦿
Armstrong, J. G.	2011																											⦿
Bales, G. T.	1996																											⦿
Boccardo, F.	2002																											⦿
Boccardo, F.	1993																											⦿
Bolla, M.	2009																											⦿
Botto, H.	1989																											⦿
Brisset, J. M.	1987																											⦿
Bruun, E.	1996																											⦿
Burns-Cox, N.	2002																											⦿

Citrin, D. L.	1991																											⊙
Crawford, E. D.	1989																											⊙
D'Amico, A. V.	2008																											⊙
Denis, L. J.	1998																											⊙
Dijkman, G. A.	1997																											⊙
Eisenberger, M. A.	1998																											⊙
Hussain, M.	2013																											⊙
Irani, J.	2008																											⊙
Iversen, P.	2010																											⊙
Jones, C. U.	2011																											⊙
Kaisary, A. V.	1991																											⊙
Kotake, T.	1999																											⊙
Manikandan, R.	2005																											⊙
Mottet, N.	2012																											⊙
Navratil, H.	1987																											⊙
Organ, M.	2013																											⊙
Ostri, P.	1991																											⊙
Parmar, H.	1988																											⊙

Pavone-Macaluso, M.	1986																											⦿
Sharifi, R.	1985																											⦿
Thorpe, S. C.	1996																											⦿
Tyrrell, C. J.	2000																											⦿
Wirth, M. P.	2004																											⦿
de Bono, J. S.	2011																										⦿	
Ryan, C. J.	2013																										⦿	
Sun, Y.	2016																										⦿	
Attard, G.	2017																										⦿	
Trachtenberg, J.	2002																								⦿			
McLeod, D.	2001																								⦿			
Garnick, M. B.	2004																								⦿			
Garnick, M.	2004																								⦿			
Debruyne, F.	2006																								⦿			
Debruyne, F. M.	2004																								⦿			
Klotz, L.	2014																								⦿			
Albertsen, P.	2013																								⦿			
Albertsen, P.C.	2013																								⦿			

Crawford, E.D.	2013																							⊙			
Shore, N.	2013																							⊙			
Mason, M. D.	2014																							⊙			
Mason, M.	2012																							⊙			
Axcrona, K.	2012																							⊙			
Tombal, B.	2009																			⊙								
Iversen, P.	2011																			⊙								
CA 7%																												
CCA 3.8 %																												

Supplement Table 4. Citation matrix of all included systematic reviews and meta-analysis and Overlapping test for OS outcome

Individual study		SRMAs																			
		2021	2021	2020	2020	2020	2020	2020	2019	2018	2018	2018	2018	2018	2017	2017	2016	2016	2016	2016	2016
		Ferro, M.	Wang, L.	Sathianathen, N.J.	Chen, J.	Wang, Y.	Nunno, V.D.	Sathianathen, N.J.	Landre, T.	Vale, C.L.	Feyerabend, S.	Wallis, C.J.D.	Kassem, L.	Tan, P.S.	Aoun, F.	Rydzewska, L.H.M.	Ramos-Esquivela, A.	Botrel, T.E.A.	Lei, J.H.	Tucci, M.	Vale, C.L.
Hoyle, A. P.	2019	⊙	⊙		⊙			⊙													
Sydes, M. R.	2018	⊙	⊙		⊙						⊙										
James, N. D.	2017		⊙	⊙	⊙	⊙	⊙	⊙	⊙	⊙	⊙	⊙	⊙	⊙	⊙	⊙					
Kyriakopoulos, C. E.	2018	⊙	⊙				⊙				⊙										
Gravis, G.	2016	⊙	⊙	⊙	⊙	⊙	⊙			⊙	⊙	⊙	⊙	⊙	⊙			⊙			
Clarke, N. W.	2019	⊙	⊙			⊙															
James, N.	2020	⊙																			
Fizazi, K.	2019	⊙	⊙	⊙		⊙		⊙													
Chi, K. N.	2019	⊙	⊙	⊙	⊙	⊙	⊙														

Davis, I. D.	2019	⊙		⊙	⊙	⊙	⊙														
Gravis, G.	2013		⊙	⊙	⊙		⊙						⊙	⊙			⊙	⊙	⊙	⊙	⊙
Sweeney, C. J.	2015		⊙	⊙	⊙	⊙	⊙		⊙		⊙	⊙	⊙	⊙	⊙		⊙	⊙		⊙	⊙
Morgans, A. K.	2018		⊙																		
James, N. D.	2016		⊙	⊙	⊙		⊙		⊙	⊙	⊙	⊙	⊙	⊙	⊙		⊙				⊙
Fizazi, K.	2017		⊙	⊙	⊙		⊙	⊙	⊙	⊙	⊙	⊙	⊙	⊙	⊙	⊙					
Chi, K. N.	2018		⊙																		
Fukasawa, S.	2018		⊙																		
Agarwal, N.	2019		⊙																		
Armstrong, A. J.	2019		⊙	⊙	⊙																
Davis, I. D.	2019		⊙																		
Fizazi, K.	2018							⊙													
Fizazi, K.	2013							⊙													
Fizazi, K.	2017							⊙													
James, N.D.	2017							⊙													
Fizazi, K.	2018							⊙													
Sweeney, C.	2016									⊙											
Gravis, G.	2018										⊙										
Patrick-Miller, L.J.	2016										⊙										

Sweeney, C.	2014																	⊙	⊙		
Rajan, P.	2015												⊙								
Gravis, G.	2015																	⊙		⊙	⊙
James, N.D.	2015																	⊙		⊙	
James, N.D.	2015																	⊙			
CA 21%																					
CCA 16.4%																					
